# Not-So-Rare Defects of RBC Lipidic Composition: Four New Cases of Flippase Deficiency Due to *ATP11C* Mutations

**DOI:** 10.3390/ijms26167722

**Published:** 2025-08-10

**Authors:** Elisa Fermo, Elena Trombetta, Anna Paola Marcello, Cristina Vercellati, Giulia Maria Ferrari, Anna Zaninoni, Valentina Brancaleoni, Elena Di Pierro, Sara Beneventi, Marta Tornese, Bruno Fattizzo, Tommaso Casini, Paola Corti, Paola Bianchi

**Affiliations:** 1 Hematology, Physiopathology of Anemia Unit, Fondazione IRCCS Ca’Granda Ospedale Maggiore Policlinico, 20122 Milano, Italy; anna.marcello@policlinico.mi.it (A.P.M.); cristina.vercellati@policlinico.mi.it (C.V.); anna.zaninoni@policlinico.mi.it (A.Z.); sara.beneventi@policlinico.mi.it (S.B.); bruno.fattizzo@policlinico.mi.it (B.F.); paola.bianchi@policlinico.mi.it (P.B.); 2 Flow Cytometry Service, Clinical Pathology, Fondazione IRCCS Ca’ Granda Ospedale Maggiore Policlinico, 20122 Milano, Italy; elena.trombetta@policlinico.mi.it (E.T.); marta.tornese@policlinico.mi.it (M.T.); 3 Pediatrics, Fondazione IRCCS San Gerardo dei Tintori, 20900 Monza, Italy; giuliamaria.ferrari@irccs-sangerardo.it (G.M.F.); paolaconsuelo.corti@irccs-sangerardo.it (P.C.); 4 SC Medicina ad Indirizzo Metabolico, Fondazione IRCCS Ca’ Granda Ospedale Maggiore Policlinico, 20122 Milano, Italyelena.dipierro@policlinico.mi.it (E.D.P.); 5 Department of Oncology and Hemato-Oncology, University of Milan, 20122 Milan, Italy; 6 Department of Haematology-Oncology, Meyer University Children’s Hospital, 50139 Firenze, Italy; tommaso.casini@meyer.it

**Keywords:** flippase, phosphatidylserine exposure, *ATP11C*, hemolysis

## Abstract

Adenosine Triphosphatase (ATPase) Phospholipid Transporting 11C gene (*ATP11C*), located on the X chromosome, encodes the major phosphatidylserine flippase in human erythroid cells. Only five patients have so far been reported with defective *ATP11C*, displaying mild hemolytic anemia and reduced flippase activity. In this study, we report four Italian male patients in three unrelated families with novel private mutations in the *ATP11C* gene, resulting in impaired flippase activity associated with mild/compensated hemolytic anemia. The decreased flippase activity was measured as % of phosphatidylserine internalization over time and ranged after 20 min incubation from 5% to 18.6% in all patients, regardless of the type of molecular defect. Flippase activity was also tested in healthy controls, ranging from 43% to 62% in both males and females. This measurement appears to be a useful tool for hypothesizing *ATP11C* abnormalities in male subjects with mild compensated hemolysis, prior to next generation sequencing (NGS) analysis. Although rare, *ATP11C* mutations may be underrecognized, and therefore should be suspected and investigated in male patients presenting with subtle hemolytic signs or symptoms.

## 1. Introduction

Congenital hemolytic anemias (CHAs) represent a highly heterogeneous group of rare inherited disorders resulting from abnormalities in red blood cell (RBC) membrane structure, metabolic pathways, transport functions, or erythropoiesis. Due to their genetic and clinical heterogeneity, as well as their rarity, CHAs remain challenging to diagnose and manage. Currently, there is a lack of standardized and widely accessible diagnostic tools, stratification systems, and therapeutic algorithms. Moreover, the pathophysiological mechanisms underlying certain atypical forms are still poorly understood.

All CHAs are characterized by hemolysis and anemia of variable severity, ranging from transfusion-dependent cases to compensated forms. Common clinical features include jaundice, splenomegaly, and in some instances iron overload. Disease progression is variable but may involve worsening anemia and an increasing requirement for transfusions. Long-term complications include iron overload, splenomegaly, impaired growth, and end-organ damage, all of which may significantly impair quality of life. Conventional management strategies, such as splenectomy and chronic transfusion therapy, carry the risk of adverse effects, particularly iron overload [1]. However, recent molecular insights have paved the way for the development and approval of targeted pharmacological therapies, including agents targeting iron metabolism and gene therapy approaches [2]. The laboratory diagnostic workflow for CHAs relies on a stepwise approach involving a battery of functional assays to evaluate erythropoiesis, RBC membrane integrity, and red cell metabolism. Initial investigations typically include markers of hemolysis, peripheral blood smear analysis, the eosin-5′-maleimide (EMA) binding test, osmotic gradient ektacytometry, and a panel of enzymatic assays. However, many of these tests are available only in specialized reference laboratories.

CHAs are caused by pathogenic variants in more than 60 genes involved in erythropoiesis and RBC structure. The genotype–phenotype correlation is often complex and unpredictable. The advent and continuous advancement of next-generation sequencing (NGS) technologies have revolutionized the diagnostic landscape of CHAs, enabling the identification and molecular characterization of ultra-rare disorders and novel genes associated with hereditary hemolytic anemia [3].

Recently, it has been hypothesized that abnormalities in RBC lipidic composition may also interfere with the stability of the RBC membrane, with premature erythrocyte sequestration and eryptosis, and new genes involved in RBC lipidic composition have been reported to be associated with increased hemolysis. The RBC lipid bilayer is composed of equal molar proportions of cholesterol and phospholipids. While cholesterol seems to be distributed equally between the two leaflets, phospholipids are asymmetrically distributed. Phosphatidylcholine (PC) and sphingomyelin are predominantly located in the outer monolayer and exposed to the cell surface, and phosphatidylethanolamine (PE) and phosphatidylserine (PS) are confined to the inner monolayer [4,5]. Phospholipid asymmetry is maintained by different types of energy-dependent and energy-independent transport proteins, in particular the flippases family (from the outer to the inner monolayer), the floppases (from inner to outside against gradient concentration) and the calcium (Ca^2+^)-dependent scramblases transport system (bi-directional movement according to their concentration gradients in an energy-independent manner) [6,7]. This asymmetry is crucial for erythrocyte survival and death; in fact, senescent RBCs show decreased flippase activity, and the consequent surface exposure of PS is a signal for eryptosis, a mechanism for the RBC clearance from blood circulation [8,9,10,11,12]. PS is prematurely exposed in various RBC disorders, including sickle cell disease and thalassemia, contributing to the pathophysiology of these diseases [13,14]. Adenosine Triphosphatase (ATPase) Phospholipid Transporting 11C gene (*ATP11C*), localized on chromosome X, encodes the major PS flippase of human RBCs [15]. Only five cases have been reported so far, with defective *ATP11C* displaying mild hemolytic anemia [15,16,17,18,19].

In this study, we report four Italian male patients with flippase deficiency due to novel mutations in the *ATP11C* gene and associated with mild/compensated hemolytic anemia.

## 2. Results

### 2.1. Case Reports

Family 1. F1-P1 is a 16-year-old Italian boy. Parents are not consanguineous, with an unremarkable medical history. He was born at 40 + 2 weeks after a clinically normal pregnancy; neonatal jaundice was treated by phototherapy, and subsequently he had normal height and weight growth and psychomotor development. Laboratory and clinical investigation following the detection of scleral jaundice revealed compensated hemolysis (Hb 14.8 g/dL, reticulocytes 4%, unconjugated bilirubin 1.81 mg/dL) and splenomegaly (13.6 cm). Urobilinogen was also increased, further confirming hemolysis (4 mg/dL (n.v. 0.0–0.2). A 9-year-old brother (F1-P2) with no clinical symptoms was also studied.

Family 2. The propositus (F2-P1) is a 7-year-old boy, the second child, and was born at term from non-consanguineous Italian parents. During infancy he had recurrent asthmatic bronchitis. Mild jaundice was first noticed by his pediatrician during one of these episodes, leading to investigations for possible hemolytic anemia. His spleen was enlarged even though the abdominal ultrasound showed interpolar diameter within normal limits for age. Laboratory parameters showed the following: Hb 11.9 g/dL, reticulocytes 380 × 10^9^/L, increased unconjugated bilirubin (maximum total level 4.2 mg/dL, unconjugated 2.5 mg/dL) and lactic dehydrogenase (LDH) (464 IU/L, about twice the upper limit of normality), and reduced haptoglobin (<7 mg/dL). Peripheral blood smear showed mild anisopoikilocytosis with rare ovalocytes and spherocytes; the study for abnormal hemoglobins and the glucose 6 phosphate dehydrogenase (G6PD) enzyme assay were normal.

At the age of 8 years, during fever with a Parvovirus B19 infection, the child experienced severe anemia (Hb 5.3 g/dL) and reticulocytopenia (reticulocytes 0.4 × 10^9^/L), which required red blood transfusions and concomitant reduction of leukocyte (2.0 × 10^9^/L) and platelet (84 × 10^9^/L) levels. In 4–5 days, the aplastic crisis was solved with a complete recovery of the blood series. Due to suspicion of a mild form of mild hereditary spherocytosis, the patients underwent further investigations.

Family 3. F3-P1 is a young man, 19 years old, born at term with neonatal jaundice and need for phototherapy. At the age of 14 he underwent cholecystectomy. Laboratory parameters showed the following: Hb 14.4 g/dL, reticulocytes 188 × 10^9^/L, and increased bilirubin (5.1 mg/dL, unconjugated 2.5 mg/dL, *UGTA1* genotype 6/7), with normal LDH (108 IU/L) and low–normal haptoglobin (7.7 mg/dL). The peripheral blood smear showed mild anisopoikilocytosis with rare ovalocytes and spherocytes; the test for abnormal hemoglobin and the G6PD enzyme assay were negative.

### 2.2. Laboratory Investigations

**Figure 1:**
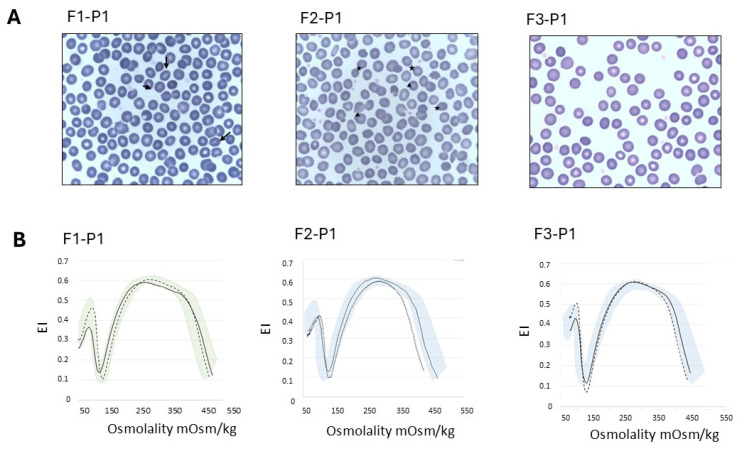
(**A**) Peripheral blood smear of patients F1-P1, F2-P1 and F3-P1. May–Grunwald–Giemsa staining, 100×. Stomatocytes are indicated with black arrows, and spherocytes and mushrooms cells are indicated with stars and triangles, respectively. (**B**) Ektacytometric Osmoscan analysis showing normal profile in patients F1-P1 and F3-P1, and reduced area (AUC) and slightly increased Omin in patient F2-P2 with concomitant hereditary spherocytosis. Dashed curve represents the patient; solid curve represents the control of the day; colored areas represent the area covered by normal controls. F1-P1: Family 1, Patient 1; F2-P1: Family 2, Patient 1; F3-P1: Family 3, Patient 1.

Hematologic and laboratory data of the probands and their family members at the time of the study are reported in Table 1 and in Figure 1.

F1-P1 showed normal osmotic fragility tests, EMA binding test, and Osmoscan curve, normal membrane protein content when analyzed with Sodium-Dodecyl-Sulfate Polyacrylamide Gel Electrophoresis (SDS-PAGE) and normal RBC enzyme activities. The RBC morphology showed mild anisopoikilocytosis with 8% of stomatocytes (Figure 1A). NGS analysis showed the presence of two novel variants (NM_001010986.3: c.828C>G (p.Asn276Lys) and c.892T>G (p.Tyr298Asp)) in the *ATP11C* gene. No other pathogenic variants that could explain the clinical phenotype were identified. As the *ATP11C* gene is located on chromosome X, the variants were found in the patient in the hemizygous state. In silico analysis suggested both mutations as variants of unknown significance (VOUS): p.Asn276Lys (located in exon 10; Protein Variation Effect Analyzer (PROVEAN) −5.32, Sorting Intolerant From Tolerant (SIFT) 0.001, supporting pathogenic PP3; Combined Annotation Dependent Depletion (CADD) Score predicted uncertain 24.299); p.Tyr298Asp (located in exon 11; PROVEAN −5.32, SIFT 0.001, supporting pathogenic PP3; CADD predicted uncertain, score 32). Both variants were also present in the healthy mother at the heterozygous level and transmitted *in cis*; therefore, it is not possible to ascertain their independent contribution. The family study revealed the presence of the variants in the clinically unaffected brother, who was consequently investigated for anemia and flippase activity.

F2-P1 showed normal osmotic fragility test results, normal membrane protein content when analyzed by SDS-PAGE analysis and normal RBC enzyme activities. Anisopoikilocytosis with rare spherocytes and rare mushroom cells was detected at RBC morphology. The EMA-binding test showed a 16% decrease in fluorescence and the Osmoscan curve was only slightly abnormal (Figure 1B). NGS analysis showed the presence of an hemizygote 13nt frameshift insertion (NM_001010986.3: c.1349_1350 insATATTTTGACATA; p.Leu451Tyrfs*9) in exon 13 of the *ATP11C* gene. The variant was predicted as VOUS at in silico analysis, although resulting in a truncated protein (rs1436217707, gnomAD ƒ = 0.000000917; PSV1 strong, PM2 supporting).

In addition, the NGS study revealed the presence of a novel variant in the *ANK1* gene (NM_000037.4: c.224C>T; pThr75Ile), also predicted as VOUS at in silico analysis (exon 3; PROVEAN −5.38, SIFT 0.00, PP3 supporting; CADD predicted pathogenic supporting, 27.2).

A co-presence of a membrane defect could explain the observation of rare spherocytes at blood film analysis, the low decrease in the EMA-binding test results, and the borderline Osmoscan curve (Figure 1B).

The *ATP11C* variant was identified in the healthy mother. None of the parents showed the presence of the *ANK1* variant, which was consequently considered as likely pathogenic with a de novo transmission.

**Figure 2:**
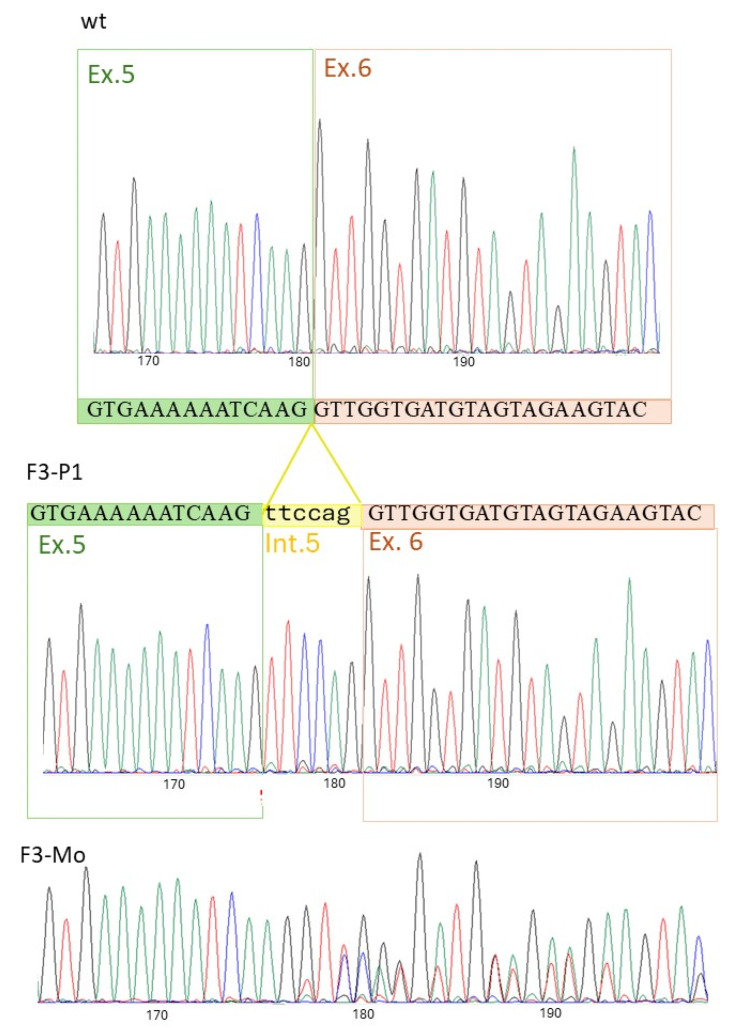
Sanger sequencing of the RT-PCR encompassing exons 5–8 of the *ATP-11C* transcript in Family 3. The sequence shows the insertion of a fragment of 6bp corresponding to the terminal portion of intron 5 in the proband (F3-P1).

F3-P1 had normal results for the osmotic fragility tests, EMA binding test, Osmoscan curve, and membrane protein content when analyzed by SDS-PAGE. RBC morphology showed 5% echinocytes, 2% spherocytes and rare ovalocytes. A variant in intron 5 of the *ATP11C* gene was detected by NGS (NM_001010986.3: c.436-7A>G), and, although not falling in splicing canonical positions, it was predicted to affect splicing (Splice AI: acceptor loss at position −7, D score 0.57; donor gain at position −1, D score 1; dbscSNV score (ADA): 0.9969, and CADD predicted pathogenic supporting 27.2). The variant had not been reported before, either in the literature or in GenomeAD, ExAc or ESP databases. Reverse-transcription polymerase chain reaction (RT-PCR) analysis showed, in the proband, the insertion of a fragment corresponding to the terminal portion of intron 5 (6bp: TTCCAG) into the *ATP11C* transcript. The mother was heterozygous and the father had normal results. At the protein level, the mutation resulted in the insertion of two amino acids at the junction between exons 5 and 6 (Lys145_Val146 ins PheGln) (Figure 2).

### 2.3. Flippase Activity Assay

**Figure 3:**
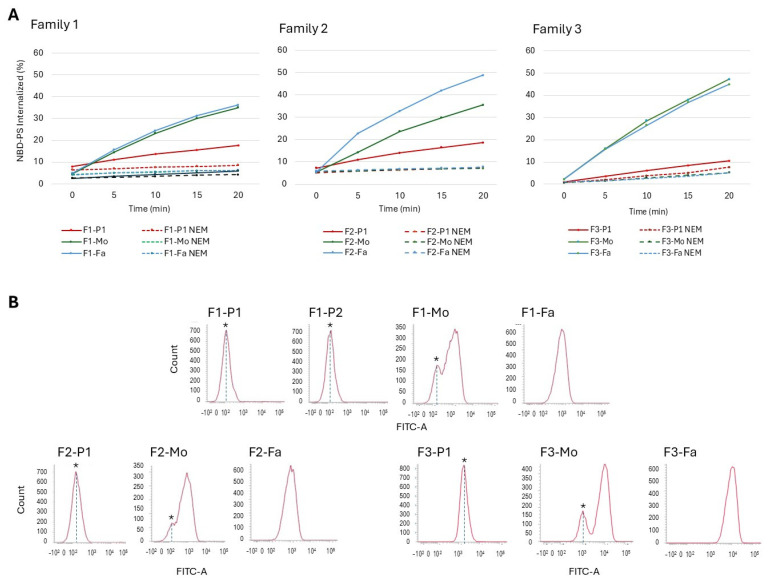
Flippase activity of RBCs of patients with *ATP11C* mutations and their relatives. (**A**) Proportion of fluorescent phosphatidylserine (NBD-PS) transported into the inner leaflet in patients and family members after 20 min incubation (MFI NBD-PS + BSA/MFI loaded NBD-PS, colored lines). Dotted lines represent blocked NBD-PS internalization after NEM treatment. Values are mean *n*  =  2 experiments for each sample. (**B**) Primary NBD-derived fluorescence data from flow cytometry. NBD-PS loaded onto erythrocyte membranes for 20 min. Stars indicate the population of the NBD-PS transported defective cells. MFI: Mean fluorescence intensity; BSA: bovine serum albumin. NEM:  mM N-ethylmaleimide; F1-P1: Family 1, Patient 1; F1-P2: Family 1, Patient 2; F1-Mo: Family 1, mother; F1-Fa: Family 1, father; F2-P1: Family 2, Patient 1; F2-Mo: Family 2, mother; F2-Fa: Family 2, father; F3-P1: Family 3, Patient 1; F3-Mo: Family 3, mother; F3-Fa: Family 3, father.

All RBCs from the four male cases carrying *ATP11C* variants (F1-P1, F1-P2, F2-P1, F3-P1) displayed a clear decrease in PS flipping activity measured as % of fluorescent PS (NBD-PS) internalized after 20 min incubation (Figure 3A): 17.6% and 5.8% of residual activity for F1-P1 and his brother with, respectively, 18.6% in F2-P1 and 10.6% in F3-P1. Healthy parents displayed variable flippase activities in the range of 35–50%. Interestingly, maternal erythrocytes of all families showed the presence of two populations (Figure 3B), possibly depending on random inactivation of the X chromosome.

Twenty healthy subjects (10 males, 10 females) were tested for flippase activity, showing a mean activity after 20 min incubation of 53% (range 43–62%), with no differences between males and females.

### 2.4. X-Chromosomal Inactivation

**Figure 4:**
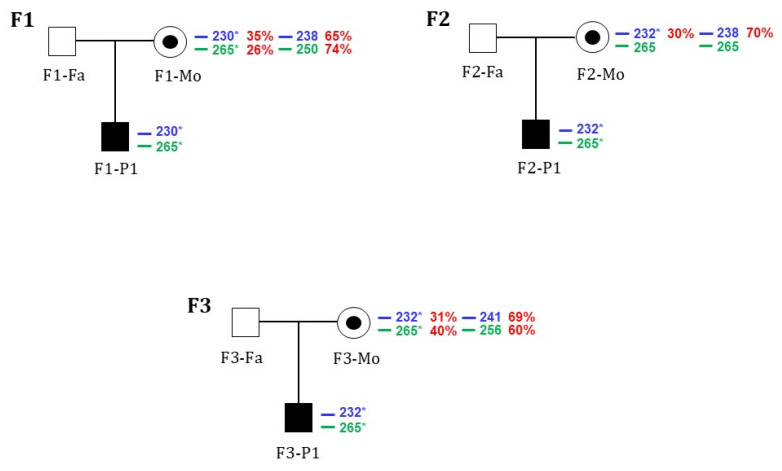
Chromosome X inactivation evaluation. The asterisk (*) indicates the X chromosome bearing the flippase variant. The percentage of X-inactivation for each allele is shown in red. The size of the AR allele is shown in blue, and the size of the *ZMYM3* polymorphisms is shown in green. Symptomatic males are shaded in black. Black dots indicate the presence of the mutation in females. F2-Mo is not informative for the *ZMYM3* allele.

We conducted methylation-based assays to test the hypothesis that X-chromosomal inactivation could influence the different expression of flippase activity in the female subjects. Fragment size analysis of the mothers allowed us to identify the size of microsatellites associated with the allele carrying the new flippase variant in each family. In Families 1 and 3, the results were comparable for both AR and *ZMYM3* assays. The *ZMYM3* assay was uninformative in Family 2 because the patient’s mother was homozygous for the polymorphic short tandem repeat (Figure 3). The heterozygous females (F1-Mo and F2-Mo, F3-Mo) showed similar mean skewed X-chromosomal inactivation patterns of the wild-type allele, with 69.5% and 70% and 64% inactivated, respectively. The mutated alleles were inactivated for about 30% in all heterozygous females, indicating that the protein is produced in both wild-type and mutated forms (Figure 4).

## 3. Discussion

The relationship between flippase activity and red blood cell membrane stability is significant, as flippases are responsible for maintaining phospholipid asymmetry, which is crucial for RBC integrity. A decrease in flippase activity results in an increased exposure of phosphatidylserine on the cell surface, which serves as a signal for macrophage-mediated clearance of aged or damaged RBCs [15], ultimately leading to programmed cell death. This process is well-documented in the context of cell aging, where flippase activity diminishes over time, causing substantial changes in lipid distribution, increased PS exposure, and influencing cellular signaling and eryptosis [10,20,21,22]. Eryptosis, in fact, is a type of regulated cell death of mature erythrocytes critically dependent on Ca^2+^ signaling and linked with cell shrinkage, membrane blebbing, and phosphatidylserine externalization, finally working to shorten the lifespan of damaged erythrocytes and to facilitate their removal from circulation, preventing hemolysis [23]. Eryptosis is a physiological process, but uncompensated eryptosis may lead to anemia. From this point of view, there is much evidence connecting increased PS exposure, eryptosis and diseases such as sickle cell disease [13,14,24] or RBC enzyme defect (G6PD deficiency) [25].

The potential connection between flippase activity, particularly regarding *ATP11C* (which encodes a key PS flippase in RBCs), accelerated eryptosis and possibly anemia, is plausible, with some functional evidence available. For example, mutations in Atp11c in mice impair the hepatic uptake of organic anions, leading to hyperbilirubinemia and hypercholanemia [26] as well as altered erythrocyte shape, anemia, and reduced erythrocyte lifespan [27].

**Figure 5:**
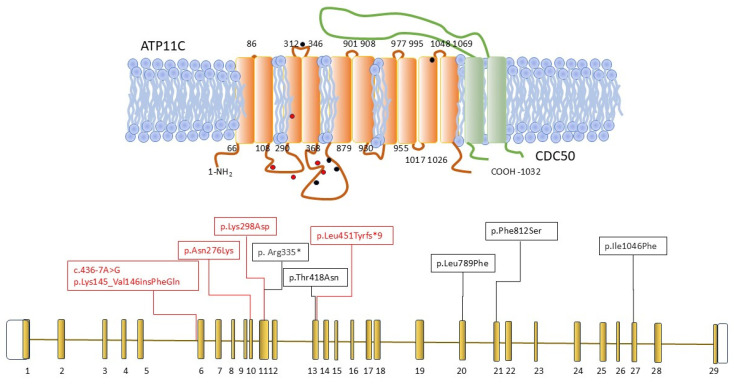
Linear schematic representation of the *ATP11C* gene and proteins (ATP11C, orange and CDC50, green). All the variants identified in this study and their positions are shown in the red boxes and circles. Variants reported in the literature as indicated in black.

Despite this, the number of patients with hemolytic anemia associated with mutations in this gene remains surprisingly limited. Since its first description about a decade ago, only five pathogenic variants have been reported in four unrelated cases: p.Thr418Asn [15,28], p.Leu789Phe [18], p.Phe812Ser [16], p.Ile1046Phe [19], and the nonsense variant p.Arg335* [17] (Table 2 and Figure 5).

One possible explanation for this observation could be the rarity of the disease, or it may be attributed to technical aspects such as the exclusion of the *ATP11C* gene from commonly used targeted panels or exome panel kits in routine clinical applications. However, the more likely explanation is the mild clinical presentation of this defect. All cases reported so far are male, due to X-linked inheritance, and typically display mild to compensated hemolysis, often only manifesting as mild reticulocytosis or slightly elevated unconjugated bilirubin, that may go unnoticed without concurrent events, such as infections [18]. The four cases described herein align perfectly with previously reported cases, investigated because of splenomegaly and reticulocytosis with compensated hemolysis. Case F1-P2 (the brother of case F1-P1) was only studied as part of a family investigation after identifying variants in the brother, revealing hemolysis. Consequently, it is possible that, although rare, *ATP11C* mutations may be underrecognized, and therefore should be suspected and investigated in male patients presenting with subtle clinical symptoms.

Although the number of variants reported so far is limited, no correlation between mutation type (missense vs. nonsense) and clinical severity has been observed; however, variants seem to cluster near functional regions, notably in the loops between TM3 and TM6 involved in lipid binding, and the interaction interface with the regulatory protein CDC50, critical for ATP11C folding and localization [29]. A clear reduction in flippase activity (from 6% to 17% after a 20 min incubation) has been noted in all reported cases. Specifically, case F3-P1, carrying a non-canonical splicing variant (c.436-7A>G), showed 90% inactivation, supporting the pathogenicity of this variant. The molecular mechanism of the variant has been elucidated, resulting in a two-amino-acid insertion of Lys145_Val146 insPheGln, possibly altering the structure of the intra-cytoplasmatic loop.

Considering that laboratory tests commonly used to detect other RBC membrane defects do not assist with a suspected flippase defect (e.g., RBC morphology, osmotic fragility tests, EMA binding test, ektacytometry), the measurement of flippase activity, evaluated by flow cytometry as a percentage of PS internalization after incubation with NBD-PS (as previously described by Arashiki et al., 2016, and van Dijk et al., 2023) [15,18] may be a helpful tool for hypothesizing *ATP11C* abnormalities before NGS analysis. In this context, we examined a control group of healthy blood donors to establish a reference range, observing no significant differences between males and females. Despite the analysis of X chromosome inactivation performed in the heterozygous mothers showing a proportion of inactivation of the mutated allele around 30%, suggestive of predominant inactivation of the normal allele, their percentage of PS internalization did not differ from the control group, except for F2-Mo, who exhibited a % of PS internalization at the lower range of healthy subjects. These data suggest that in females, a minimum residual flippase activity is still sufficient for cell functionality, not altering membrane composition and not hesitating in haemolysis.

Interestingly, patient F2-P1 presented an additional de novo *ANK1* VOUS variant, potentially resulting in a mild form of hereditary spherocytosis, which could explain the mild abnormalities observed in the EMA binding test and ektacytometry. Two other cases reported in the literature [16,19] presented with a similar condition (VOUS variants in *ANK1* gene). It is not possible to ascertain whether the co-inheritance of *ATP11C* and *ANK1* variants is just a causality; however, the concomitance of two mild defects may result in a worsening of clinical presentation during stress or infections. This was the case for patient F2-P1, whose Hb level fell down to 5.3g/dL, requiring blood transfusions in conjunction with a Parvovirus B19 infection.

## 4. Materials and Methods

### 4.1. Hematologic Studies

Peripheral blood was collected from all the patients and relatives as blood needed to be drawn for diagnostic work up. Informed consent was obtained before proceeding with the genetic analysis. In all patients, routine hematological and biochemical investigations were carried out, as follows. The RBC morphology was assessed by two independent and expert operators; red cell osmotic fragility (OF) was evaluated by a NaCl test on both fresh and incubated blood, a standard glycerol lysis test (GLT), acidified glycerol lysis test (AGLT) and Pink test [30]; and an Eosin-5-maleimide (EMA)-binding test [31] study of RBC deformability in osmotic gradient conditions (Osmoscan analysis) was carried out using LoRRca MaxSis (Laser-Assisted Optical Rotational Cell Analyzer, Mechatronics, Zwaag, The Netherlands) [32]. RBC membrane protein analyses by SDS–PAGE following Fairbanks and Laemmli methods were also performed [33].

### 4.2. Molecular Studies

The DNA sample of the probands was analyzed on a next-generation sequencing (NGS)-targeted panel containing 51 genes associated with congenital hemolytic anemias [34] (Supplemental Table S1). Libraries were obtained using a SureSelect X HS Reagent Kit (Agilent Technologies, Santa Clara, CA, USA) and sequenced on a MiSeq platform (Illumina, San Diego, CA, USA) following manufacturer instructions. Variants were identified and annotated using the Alissa Align&Call and Interpret software (https://www.agilent.com/en/product/next-generation-sequencing/ngs-data-analysis-interpretation/alissa-interpret-4301560, accessed on 6 August 2025) (Agilent Technologies). The identified variants were confirmed by Sanger sequencing (SeqStudio Genetic Analyzer, Thermo Fisher Scientific, Waltham, MA, USA) using the Big Dye Terminator Cycle Sequencing Kit (Thermo Fisher) in the probands and family members.

RT-PCR was performed to investigate possible effects of the c.436-7A>G variant on the *ATP11C* splicing pattern in Family 3. Total RNA was isolated from the peripheral blood of patients, parents and a healthy control, using the LEV simplyRNA Blood Kit for Maxwell^®^16 (Promega Corporation, Madison, WI, USA), according to the protocol described in Fiorentino et al. [35]. Briefly, 250 ng of total RNA was reverse-transcribed using the Superscript IV VILO Master Mix (Thermo Fisher) following the protocol supplied with the kit. The region spanning from exons 5 to 8 of the *ATP11C* gene was amplified by PCR using the forward and reverse primers Fw-CTTCCTTGTACAGGTCACAGT and Rev-GTCCCAAAGACCTGGCAACA, and directly sequenced.

### 4.3. Measurement of Flippase Activities in Human RBCs

Flippase activity was determined by measuring PS internalization on purified RBCs using flow cytometry, in accordance with previously reported methods [15,18].

Briefly, 10 μL of 0.1 mg/mL Fluorescent PS (16:6-06:0 NBD-PS, Avanti Polar Lipids, Inc., Albaster, AL, USA) was added to 1 mL of washed erythrocytes resuspended at 5% hematocrit in PBS with 0.2% glucose (PBS-G); samples were incubated for 0–20 min at 37 °C, and every 5 min, 20 μL of the erythrocyte suspension was washed with 1 mL PBS-G with 1% bovine serum albumin (BSA; Sigma-Aldrich, St. Louis, MA, USA) to remove remaining NBD-PS, such that the remaining fluorescence represented PS that flipped to the inner leaflet. To measure loaded NBD-PS after the 20 min incubation, incubated erythrocytes were also washed with PBS-G in the absence of BSA. NBD-derived fluorescence was measured in duplicate by flow cytometry (BD FACSLyric, Becton Dickinson, Franklin Lakes, NJ, USA), acquiring 20000 RBC per sample. Median fluorescence intensities (MFI) of the FITC channel were used to obtain the % of the NBD-PS internalized at the due time, by dividing the MFI from each condition with that of the loaded NBD-PS. To verify the basal level of flippase-independent flipping activity, the cell suspension was also treated with 5 mM N-ethylmaleimide (NEM) in PBS-G for 20 min at 37 °C, which irreversibly and non-specifically inactivates flippases. Following the same procedure, the flippase activity was measured at 20 min in 20 blood donors (10 males, 10 females) to create the expected range in healthy subjects.

### 4.4. X-Chromosomal Inactivation

X-chromosomal inactivation was assessed using methylation-based analysis of the human Androgen Receptor (AR) and zinc-finger MYM type 3 (ZMYM3) genes, as previously described [36], with minor modifications. Briefly, 250 ng DNA samples from each heterozygous female were digested with 20U HpaII and 20U HhaI (Promega). Digested and undigested DNA (50 ng) was amplified in separate tubes using FAM and HEX-labelled forward and reverse primers (0.5 µM) in a reaction containing 1x Q5^®^ High-Fidelity Master Mix (New England Biolabs, Ipswich, MA, USA). Primer sequences are available upon request. The PCR conditions were as follows: 98 °C for 30 s, then 35 cycles of 98 °C for 5 s, 66 °C (AR) or 69 °C (ZMYM3) for 10 s, and 72 °C for 30 s. PCR products were electrophoresed on an automatic sequencer (SeqStudio, Thermo Fisher Scientific) for fragment separation. Fragment analysis of the digested (d) and undigested (u) samples was performed using Cloud Microsatellite Analysis Software (Thermo Fisher Scientific). The degree of skewing for each gene was calculated as follows; (AlleleA_d/AlleleA_u)/(AlleleA_d/AlleleA_u + AlleleB_d/AlleleB_u), where AlleleA_d and AlleleB_d represent the peak heights of the digested sample, while AlleleA_u and AlleleB_u correspond to the peak heights of the undigested sample [35]. Results are shown as the mean of AR and ZMYM3 percentage of inactivation.

### 4.5. Materials

Details of the methods performed are reported in the corresponding references, and chemicals and kits used for the analysis are reported in Supplementary Table S2.

## 5. Conclusions

In conclusion, we report four new young patients with flippase defects due to different mutations in the *ATP11C* gene. The molecular defects appear to be heterogeneous (with all identified variants being private), but are consistently associated with a mild phenotype. Nonetheless, identifying these defects and providing careful patient follow-up is crucial to monitor worsening of hemolysis during infections and to anticipate potential though currently unknown complications during aging.

## Figures and Tables

**Table 1:** Clinical, hematological, biochemical and molecular data of the patients and family members at the time of the study. # During Parvovirus B19 infection; § concomitant *ANK1* c.224C>T (p.Thr75Ile) variant;. ° Hb beta-carrier. Hb: hemoglobin; MCV: mean corpuscular volume; RDW: red cell distribution width; LDH: lactate dehydrogenase. F1-P1: Family 1, Patient 1; F1-P2: Family 1, Patient 2; F1-Mo: Family 1, mother; F1-Fa: Family 1, father; F2-P1: Family 2, Patient 1; F2-Mo: Family 2, mother; F2-Fa: Family 2, father; F3-P1: Family 3, Patient 1; F3-Mo: Family 3, mother; F3-Fa: Family 3, father.

	F1-P1	F1-P2	F1-Mo	F1-Fa	F2-P1	F2-Mo	F2-Fa	F3-P1	F3-Mo	F3-Fa	Ref. Values
Age (yr)	16	9	41	54	9	45	47	19	56	57	
Neonatal jaundice	yes	no	no	no	no	no	no	yes	no	no	
Splenomegaly	yes (13cm)	no	no	no	yes	no	no	nd	no	no	
Transfusions	no	no	no	no	yes (2 units) #	no	no	no	No	no	
Hb (g/dL)	15.6	13.3	11.2	14.9	12.2	14.4	14.7	14.4	10.7	15.3	12.1–16.7
MCV (fL)	90.7	84.5	90.3	82.6	92.6	86.6	88	94.1	65.6 °	83.7	78–99
RDW (%)	13	13.1	15.8	13	14.7	13	11.9	12.7	15.5	13.2	11.6–16.5
Reticulocytes (10^9^/L)	164	202	95	78	361	73	55	188	109	78	24–84
RBC morphology	rare spherocytes rare stomatocytes	n.a.	n.a.	n.a.	3% ovalocytes, rare spherocytes, rare mushroom cells	n.a.	n.a.	5% echinocytes 2% spherocytes rare ovalocytes	n.a.	n.a.	n.a.
Unconjugated bilirubin (mg/dL)	1.81	n.a.	n.a.	n.a.	2.56	n.a.	n.a.	5	0.88	n.a.	<1.00
Haptoglobin (mg/dL)	139	n.a.	n.a.	n.a.	<7	n.a.	n.a.	58	115	145	30–200
LDH (IU/L)	234	n.a.	n.a.	n.a.	464	n.a.	n.a.	108	158	170	125–220
Residual flippase activity (%)	17.6	5.8	35	36	18.6	35.5	48.9	10.6	47.23	44.9	43–62
*ATP11C* variant	c.828C>G; (p. Asn276Lys) c.892T>G; (p. Tyr298Asp)			wt	§c.1349_1350insATATTTTGACATA; (p.Leu451Tyrfs*9)		wt	c.436-7A>G; (Lys145_Val146 ins PheGln)		wt	

**Table 2:** *ATP11C*-deficient cases reported in the literature.

Case	Age at Diagnosis	Clinical Presentation	Hb (g/dL)	Reticulocytes (10^9^/L)	Unconj. Bilirubin (mg/dL)	ATP11C Gene Mutation	Residual Flippase Activity (%)
[15]	14	pigmented urine (4yrs) mild anemia (13yrs)	11.8	53	1	c.1253C>A (p.Thr418Asn)	3%
[17]	15	jaundice, pigmented urine	11.9	208	4	c.1003C>T (p.Arg335*)	n.a
[18]	37	mild splenomegaly, fatigue, reduced exercise capacity	13.2	194	1.8 #	c.2365C> T (p. Leu789Phe)	10.8%
[16] °	n.a	splenomegaly, compensated hemolysis	14.7	107	1.5	c.2434C>T (p.Phe812Ser)	n.a
[19] §	birth	anemia, neonatal jaundice	6	92-233	12.2	c.3136A>T (p.Ile1046Phe)	n.a

# Total bilirubin; § concomitant HS due to *ANK1* variant c.937del (p.Ala313Leufs*19); ° concomitant presence of *ANK1* VOUS c.4558G > C (p.Glu1520Gln); n.a: not available.

## Data Availability

The original contributions presented in this study are included in the article/supplementary material. Further inquiries can be directed to the corresponding author.

## Supplementary Materials

The supporting information can be downloaded at https://www.mdpi.com/article/10.3390/ijms26167722/s1.

## References

[1] Risinger M., Emberesh M., Kalfa T.A. (2019). Rare Hereditary Hemolytic Anemias: Diagnostic Approach and Considerations in Management. Hematol. Oncol. Clin. N. Am..

[2] Al-Samkari H., van Beers E.J. (2021). Mitapivat, a novel pyruvate kinase activator, for the treatment of hereditary hemolytic anemias. Ther. Adv. Hematol..

[3] Agarwal A.M., Rets A.V. (2023). Molecular diagnosis of hereditary hemolytic anemias: Recent updates. Int. J. Lab. Hematol..

[4] Manno S., Takakuwa Y., Mohandas N. (2002). Identification of a functional role for lipid asymmetry in biological membranes: Phosphatidylserine-skeletal protein interactions modulate membrane stability. Proc. Natl. Acad. Sci. USA.

[5] Zwaal R.F.A., Comfurius P., Bevers E.M. (2005). Surface exposure of phosphatidylserine in pathological cells. Cell. Mol. Life Sci..

[6] Andersen J.P., Vestergaard A.L., Mikkelsen S.A., Mogensen L.S., Chalat M., Molday R.S. (2016). P4-ATPases as phospholipid flippases—Structure, function, and enigmas. Front. Physiol..

[7] Arashiki N., Takakuwa Y. (2017). Maintenance and regulation of asymmetric phospholipid distribution in human erythrocyte membranes: Implications for erythrocyte functions. Curr. Opin. Hematol..

[8] Connor J., Pak C.C., Schroit A.J. (1994). Exposure of phosphatidylserine in the outer leaflet of human red blood cells. Relationship to cell density, cell age, and clearance by mononuclear cells. J. Biol. Chem..

[9] Boas F.E., Forman L., Beutler E. (1998). Phosphatidylserine exposure and red cell viability in red cell aging and in hemolytic anemia. Proc. Natl. Acad. Sci. USA.

[10] Seki M., Arashiki N., Takakuwa Y., Nitta K., Nakamura F. (2020). Reduction in flippase activity contributes to surface presentation of phosphatidylserine in human senescent erythrocytes. J. Cell. Mol. Med..

[11] Tkachenko A., Alfhili M.A., Alsughayyir J., Attanzio A., Al Mamun Bhuyan A., Bukowska B., Cilla A., Quintanar-Escorza M.A., Föller M., Havranek O. (2025). Current understanding of eryptosis: Mechanisms, physiological functions, role in disease, pharmacological applications, and nomenclature recommendations. Cell Death Dis..

[12] Tkachenko A. (2024). Apoptosis and eryptosis: Similarities and differences. Apoptosis.

[13] Wood B.L., Gibson D.F., Tait J.F. (1996). Increased erythrocyte phosphatidylserine exposure in sickle cell disease: Flow-cytometric measurement and clinical associations. Blood.

[14] Kuypers F.A., Yuan J., Lewis R.A., Snyder L.M., Kiefer C.R., Bunyaratvej A., Fucharoen S., Ma L., Styles L., de Jong K. (1998). Membrane phospholipid asymmetry in human thalassemia. Blood.

[15] Arashiki N., Takakuwa Y., Mohandas N., Hale J., Yoshida K., Ogura H., Utsugisawa T., Ohga S., Miyano S., Ogawa S. (2016). ATP11C is a major flippase in human erythrocytes and its defect causes congenital hemolytic anemia. Haematologica.

[16] Mansour-Hendili L., Aissat A., Badaoui B., Sakka M., Gameiro C., Ortonne V., Wagner-Ballon O., Pissard S., Picard V., Ghazal K. (2020). Exome sequencing for diagnosis of congenital hemolytic anemia. Orphanet J. Rare Dis..

[17] Zhang H.M., Yang L., Dong W., Liu Q.C. (2022). Congenital hemolytic anemia caused by a new mutation of ATP11C gene: A case report. Zhonghua Xue Ye Xue Za Zhi.

[18] van Dijk M.J., van Oirschot B.A., Harrison A.N., Recktenwald S.M., Qiao M., Stommen A., Cloos A.S., Vanderroost J., Terrasi R., Dey K. (2023). A novel missense variant in ATP11C is associated with reduced red blood cell phosphatidylserine flippase activity and mild hereditary hemolytic anemia. Am. J. Hematol..

[19] Xu W., Ma M., Zhao S., Yuan Y., Tian Z. (2023). Case of Congenital Hemolytic Anemia with ATP11C and ANK1 Variants. Children.

[20] Sai K.V., Lee JY. E. (2024). Crossing the membrane – what does it take to flip a phospholipid? Structural and biochemical advances on P4-ATPase flippases. J. Biol. Chem..

[21] Hankins H.M., Baldridge R.D., Xu P., Graham T.R. (2015). Role of flippases, scramblases and transfer proteins in phosphatidylserine subcellular distribution. Traffic.

[22] Čopič A., Dieudonné T., Lenoir G. (2023). Phosphatidylserine transport in cell life and death. Curr. Opin. Cell Biol..

[23] Föller M., Lang F. (2020). Ion Transport in Eryptosis, the Suicidal Death of Erythrocytes. Front. Cell Dev. Biol..

[24] Nader E., Romana M., Guillot N., Fort R., Stauffer E., Lemonne N., Garnier Y., Chambers Skinner S., Etienne-Julan M., Robert M. (2020). Association Between Nitric Oxide, Oxidative Stress, Eryptosis, Red Blood Cell Microparticles, and Vascular Function in Sickle Cell Anemia. Front. Immunol..

[25] Bouguerra G., Talbi K., Trabelsi N., Chaouachi D., Boudriga I., Abbès S., Menif S. (2021). Enhanced Eryptosis in Glucose-6-Phosphate Dehydrogenase Deficiency. Cell. Physiol. Biochem..

[26] Matsuzaka Y., Hayashi H., Kusuhara H. (2015). Impaired Hepatic Uptake by Organic Anion-Transporting Polypeptides Is Associated with Hyperbilirubinemia and Hypercholanemia in Atp11c Mutant Mice. Mol. Pharmacol..

[27] Yabas M., Coupland L.A., Cromer D., Winterberg M., Teoh N.C., D’Rozario J., Kirk K., Bröer S., Parish C.R., Enders A. (2014). Mice deficient in the putative phospholipid flippase Atp11c exhibit altered erythrocyte shape, anemia, and reduced erythrocyte life span. J. Biol. Chem..

[28] Arashiki N., Niitsuma K., Seki M., Takakuwa Y., Nakamura F. (2019). ATP11C T418N, a gene mutation causing congenital hemolytic anemia, reduces flippase activity due to improper membrane trafficking. Biochem. Biophys. Res. Commun..

[29] Hanayo Nakanishi H., Irie K., Segawa H., Hasegawa K., Fujiyoshi Y., Nagata S., Abe K. (2020). Crystal structure of a human plasma membrane phospholipid flippase. J. Biol. Chem..

[30] Vercellati C., Zaninoni A., Marcello A.P., Fermo E., Fattizzo B., Giannotta J.A., Bianchi P., Zanella A., Barcellini W. (2022). Changing trends of splenectomy in hereditary spherocytosis: The experience of a reference Centre in the last 40 years. Br. J. Haematol..

[31] Bianchi P., Fermo E., Vercellati C., Marcello A.P., Porretti L., Cortelezzi A., Barcellini W., Zanella A. (2012). Diagnostic power of laboratory tests for hereditary spherocytosis: A comparison study in 150 patients grouped according to molecular and clinical characteristics. Haematologica.

[32] Zaninoni A., Fermo E., Vercellati C., Consonni D., Marcello A.P., Zanella A., Cortelezzi A., Barcellini W., Bianchi P. (2018). Use of laser assisted optical rotational cell analyzer (LoRRca MaxSis) in the diagnosis of RBC membrane disorders, enzyme defects, and congenital dyserythropoietic anemias: A monocentric study on 202 patients. Front. Physiol..

[33] Mariani M., Barcellini W., Vercellati C., Marcello A.P., Fermo E., Pedotti P., Boschetti C., Zanella A. (2008). Clinical and hematologic features of 300 patients affected by hereditary spherocytosis grouped according to the type of the membrane protein defect. Haematologica..

[34] Fermo E., Vercellati C., Marcello A.P., Keskin E.Y., Perrotta S., Zaninoni A., Brancaleoni V., Zanella A., Giannotta J.A., Barcellini W. (2021). Targeted next generation sequencing and diagnosis of congenital hemolytic anemias: A three years experience monocentric study. Front. Physiol..

[35] Fiorentino V., Brancaleoni V., Granata F., Graziadei G., Di Pierro E. (2016). The assessment of noncoding variant of PPOX gene in variegate porphyria reveals post-transcriptional role of the 5’ untranslated exon 1. Blood Cells Mol. Dis..

[36] Brancaleoni V., Balwani M., Granata F., Graziadei G., Missineo P., Fiorentino V., Fustinoni S., Cappellini M.D., Naik H., Desnick R.J. (2016). X-chromosomal inactivation directly influences the phenotypic manifestation of X-linked protoporphyria. Clin. Genet..

