# Supplementary material for: Not-So-Rare Defects of RBC Lipidic Composition: Four New Cases of Flippase Deficiency Due to ATP11C Mutations
Source: Int J Mol Sci. 2025 Aug 10;26(16):7722. doi: 10.3390/ijms26167722 (PMC12387066; doi:10.3390/ijms26167722)
Supplement: Supplementary file 1 [file ijms-26-07722-s001.zip › ijms-3769101-supplementary.pdf]

Not so rare defects of RBC lipidic composition: four new cases of flippase deficiency due to ATP11C mutations.

Supplementary materials

Supplementary table 1. List of genes included in the t-NGS panel for congenital hemolytic anemia

Gene	Ref Seq.	Gene	Ref Seq.
ABCB6	NM_005689.4	HBA2	NM_000517.6
ABCG5	NM_022436.3	HFE	NM_000410.4
ABCG8	NM_022437.3	HK1	NM_000188.3
AK1	NM_000476.3	KCNN4	NM_002233.4
ALAS2	NM_000032.5	KIF23	NM_138555.4
ALDOA	NM_184041.5	KLF1	NM_006563.5
ANK1	NM_000037.4	NT5C3A	NM_016489.14
ATP11C	NM_001010986.3	PFKL	NM_001002021.3
CDIN1	NM_001130010.3	PFKM	NM_000289.6
CDAN1	NM_138477.4	PGK1	NM_000291.4
CYB5R3	NM_000398.7	PGM1	NM_002633.3
ENO1	NM_001428.5	PIEZO1	NM_001142864.4
EPB41	NM_004437.4	PKLR	NM_000298.6
EPB42	NM_000119.3	RACGAP1	NM_013277.5
G6PD	NM_001042351.3	RHAG	NM_000324.3
GATA1	NM_002049.4	SBDS	NM_016038.4
GBA	NM_001005741.3	SEC23B	NM_006363.6
GCLC	NM_001498.4	SLC11A2	NM_000617.3
GCLM	NM_002061.4	SLC25A38	NM_017875.4
GLRX5	NM_016417.3	SLC2A1	NM_006516.4
GPI	NM_000175.5	SLC4A1	NM_000342.4
GPX1	NM_000581.4	SPTA1	NM_003126.4
GSR	NM_000637.5	SPTB	NM_001355436.2
GSS	NM_000178.4	SUPT5H	NM_003169.4
HBB	NM_000518.5	TPI1	NM_000365.6
HBA1	NM_000558.5		

Supplementary table 2. List and details of chemicals and kits used in the study

Hematological studies		
**Reagent**	**Cod.**	**Test**
Eosin 5' maleimide (EMA)	cod. 63184-10 mg<br>Sigma-Aldrich	EMA-binding tests
Bovine serum albumin (BSA)	cod. A7905 100g<br>Sigma-Aldrich	Flippase activity measurment
mM N-ethylmaleimide (NEM)	cod. 34115-M<br>Sigma-Aldrich	Flippase activity measurment
Fluorescent phosphatidylserine<br>(16:0-06:0 NBD-PS)	cod. 810192P-1MG<br>Avanti Polar Lipids	Flippase activity measurment
**Molecular studies**		
Big Dye Terminator Cycle Sequencing Kit	Thermo Fisher Scientific	Sanger sequencing
SureSelect X HS Reagent Kit	cod. G9731C; 5191-4079<br>Agilent	t-NGS
HpaII<br>HhaI	cod. ER0511; ER1851<br>Promega	X-Chrom inactivation
LEV simplyRNA Blood Kit for Maxwell®16	cod. AS1310<br>Promega	RNA extraction
Q5® High-Fidelity Master Mix	cod. M0492S<br>New England Biolabs	RT -PCR
Superscript IV VILO Master Mix	Cod. 11756050<br>Thermo Fisher Scientific	RT-PCR
